# The association between childhood maltreatment and neural functioning during a working memory task in adults with internalizing disorders

**DOI:** 10.1007/s11682-026-01120-2

**Published:** 2026-03-10

**Authors:** Morgan M. Caudle, Alan N. Simmons, Raeanne C. Moore, Michael L. Thomas, Jessica Bomyea

**Affiliations:** 1 San Diego State University/ UC San Diego Joint Doctoral Program in Clinical Psychology, 6363 Alvarado Court, Suite 103, San Diego, CA 92120 USA; 2 Department of Psychiatry, University of California San Diego, Mail Code 0855, 9500 Gilman Dr, La Jolla, CA 92093-0855 USA; 3 VA San Diego Center of Excellence for Stress and Mental Health, 3350 La Jolla Village Dr, San Diego, CA 92161 USA; 4 Department of Psychology, Colorado State University, 1876 Campus Delivery, Fort Collins, CO 80523 USA

**Keywords:** Early life stress, Working memory, FMRI, PTSD, Anxiety

## Abstract

Childhood maltreatment is pervasive – with an estimated 1 in 4 children experiencing abuse or neglect at some point in their lifetime. Exposure to childhood maltreatment has been associated with detrimental neurodevelopmental changes to brain structure and function in stress-sensitive regions and regions with extended developmental periods. Such regions include the frontoparietal regions (e.g., middle frontal gyrus and dorsolateral prefrontal cortex), which are vital for cognitive processes, including working memory; however, few studies have investigated how childhood maltreatment impacts the neural substrates implicated in supporting cognitive functioning. The present study investigated the potential influence of childhood maltreatment on neural functioning in adults (*n* = 111; aged 34.53 ± 9 years) diagnosed with internalizing disorders, with varied levels of childhood maltreatment severity, who completed a working memory task while undergoing functional magnetic resonance imaging. Voxel-wise activation data were extracted through an a-priori defined mask of regions relevant to working memory to evaluate neural activity during the task. Linear regression analyses revealed that childhood maltreatment severity was negatively associated with left dorsolateral prefrontal cortex activation during the working memory task, controlling for current symptoms of psychopathology. Childhood maltreatment was also associated with poorer working memory task performance. These findings suggest that childhood maltreatment severity is associated with decreased dorsolateral prefrontal cortex engagement during working memory function in a transdiagnostic sample. These results further the understanding of factors influencing differences in cognitive functioning and, thus, symptom presentation in clinical populations.

**Supplementary Information:**

The online version contains supplementary material available at 10.1007/s11682-026-01120-2.

## Introduction

Childhood maltreatment (CM) is highly prevalent, with an estimated one in seven children experiencing abuse or neglect in the past year (“Child maltreatment,” 2016; Lippard & Nemeroff, 2020). Exposure to CM has been causally linked with the development of psychiatric illness; however, it is also thought to lead to unique adverse clinically-relevant outcomes (Baldwin et al., 2023; Teicher et al., 2016), such as increased severity of psychopathology, poorer treatment response, and worse cognitive functioning (Curran et al., 2018; Goltermann et al., 2021; Kuzminskaite et al., 2022; Lippard & Nemeroff, 2020; Nanni et al., 2012; Nelson et al., 2017). CM has also been associated with neural alterations in regions implicated in affective and cognitive processing which may underly and lead to heterogeneity within diagnostically similar individuals (Teicher et al., 2016).

CM may exert its detrimental effects via stress induced neural changes. Exposure to CM is one type of stressor that may lead to developmental changes. Chronic or repeated exposure to stress significantly impacts neural regions developing during the exposure to the stress (Lupien et al., 2009). Stress may be particularly problematic while regions are developing and thereby more vulnerable to slowed growth, altered synaptic organization, or neurotoxicity during stress exposure (Andersen & Teicher, 2008; Lupien et al., 2009).

Despite the wealth of evidence pointing to CM’s association with disturbance in affective processes (Hanson et al., 2016; Morey et al., 2016; Teicher et al., 2016), less is known about how CM might impact non-affective processing, such as shifting attention and maintaining working memory (WM) (Gratton et al., 2017; Miyake et al., 2000). Neural regions implicated in supporting these processes are rooted in the frontoparietal network (e.g., dorsolateral prefrontal cortex (dlPFC), dorsal anterior cingulate (dACC), inferior frontal gyrus (IFG), and posterior parietal cortex (Banich et al., 2009; D’Esposito & Postle, 2015; Niendam et al., 2012). Specifically, regions vital for WM function (e.g., prefrontal and posterior parietal cortex) may be particularly at risk to childhood stress because these regions undergo prolonged neurodevelopment into late adolescence (Gogtay et al., 2004; Luciana et al., 2005; Paus, 2001; Sowell et al., 2001); Yet, the relationship between CM and neural functioning underlying working memory remains relatively understudied.

CM is associated with poorer performance on neuropsychological WM tasks (Cowell et al., 2015; DePrince et al., 2009; Lynch & Widom, 2022; Masson, Bussieres, East-Richard, A, & Cellard, 2015; Mercier et al., 2018) and provisional evidence points to alterations in neural regions implicated in cognitive processing. In healthy controls, CM has been associated with decreased neuronal activity in the prefrontal cortex and parietal regions during tasks designed to probe WM (Hallowell et al., 2019; Philip et al., 2013, 2016; Raine et al., 2001). Yet, there is a paucity of research examining the relationship between CM and neural functioning during non-affective WM tasks in populations with internalizing disorders; internalizing disorders, such as anxiety, depression, and post-traumatic stress disorder (PTSD) are highly prevalent in the general population and even more prevalent among individuals with CM (Moussavi et al., 2022; Rapsey et al., 2019). Extant research suggests that individuals with major depressive disorder (MDD) and CM exhibited greater activity in the dlPFC than those with MDD but without CM (Miller et al., 2015), in contrast to previous observations of decreased neural activity in healthy controls (Hallowell et al., 2019; Philip et al., 2013, 2016; Raine et al., 2001). The potential unique influence of CM on non-affective cognition-related neural activity in individuals with anxiety and post-traumatic stress disorders remains relatively unexplored.

To address this gap, the present study aimed to investigate associations between CM and neural activity during an in-scanner WM capacity task in adults diagnosed with mood, anxiety, or traumatic stress disorders, i.e., internalizing disorders. We hypothesized that greater CM would be associated with poorer performance on the WM task and increased neural activity in frontoparietal regions. We examined effects, controlling for current symptoms of internalizing disorders because the severity of these symptoms has been previously associated with altered neural activity during WM tasks. Given that many studies do not account for the potential influences of CM on neural functioning, if unique associations of CM with neural functioning do emerge, these results may have implications for future neuroimaging research broadly.

## Methods

### Study design

Participants (*n* = 111, 65 female), aged 34.53 ± 9 years, in the present analysis were drawn from two separate clinical trials, that had recruited treatment-seeking individuals with post-traumatic stress, anxiety, and/or mood disorders, aiming to investigate the effects of a computerized WM training intervention (see (Bomyea et al. 2025a, b). Participants in these clinical trials were recruited via flyers in mental health and primary care clinics, digital advertisements, and referrals from mental health and primary care clinics. The present analysis included all participants who had completed a WM task while undergoing fMRI at baseline assessment. The primary differences between studies were that dataset 1 (*n* = 47) included Veterans with a primary diagnosis of PTSD, whereas dataset 2 (*n* = 64) included participants diagnosed with post-traumatic stress, anxiety, and/or mood disorders (see supplement for full inclusion exclusion criteria and Table 1 for demographic and clinical characteristics of participants between datasets). Prior to enrollment, all participants provided written informed consent and the protocols were approved by the UC San Diego Institutional Review Board (IRB) or VA San Diego Healthcare System IRB.

**Table 1:** Demographics and clinical characteristics of all participants and within each dataset. ANOVA was used to compare continuous measures, and Chi-square was used to compare categorical measures (i.e., sex and count of individuals designated as early life stress (ELS) positive) between datasets

*N*	All Participants mean (SD)	Dataset 1 mean (SD)	Dataset 2 mean (SD)	Statistic
	111	47	64	
Sex: n female (% female)	67 (61%)	16 (34%)	51 (80%)	*χ*2(1) = 23.597, *p* <.001
Age (years)	34.53 (9)	37.06 (9.25)	32.67 (8.41)	*F*(1, 109) = 6.786, *p* =.01
Education n (%)				
High School Diploma/GED	8 (7.2%)	6 (12.8%)	2 (0.031)	
Some Post High School Education	24 (21.6%)	16 (34%)	8 (12.5%)	
Technical School Certificate	13 (11.7%)	11 (23.4%)	2 (3.1%)	
2-years of College	18 (16.2%)	13 (27.7%)	5 (7.8%)	
4-years of college	30 (27%)	0 (0%)	30 (46.9%)	
Graduate or Professional Study	16 (14.4%)	0 (0%)	16 (25%)	
Hispanic n (%)	34 (30.6%)	14 (29.8%)	20 (31.3%)	*χ*2(1) = 0.869 *p* =.519
Race n (%)				
Native American/Alaskan Native	8 (7.3%)	2 (4.3%)	6 (9.4%)	
Asian	15 (13.5%)	3 (6.4%)	12 (18.8)	
Black	6 (5.4%)	3 (6.4%)	3 (4.7%)	
Native Hawaiian/Pacific Islander	2 (0.018)	1 (2.1%)	1 (1.6%)	
White	78 (0.703)	30 (63.8%)	48 (75%)	
Unknown/Decline	3 (0.027)	3 (6.4%)	0 (0%)	
ELS n (% Positive)	65 (0.59)	32 (68%)	33 (52%)	*χ*2(1) = 3.049, *p* =.081
Continuous Measures Mean (SD)				
CTQ	54.95 (16.47)	61.47(18.4)	50.16 (13.1)	*F*(1, 109) = 14.33, *p* <.001
CTQ Emotional Abuse	12.57 (6.17)	12.23(6.16)	12.81 (6.22)	*F*(1, 109) = 0.236, *p* =.628
CTQ Emotional Neglect	13.16 (4.99)	12.57(6.17)	12.66 (4.31)	*F*(1, 109) = 1.56, *p* =.214
CTQ Physical Abuse	8.89 (4.8)	10.26(5.78)	7.89 (3.66)	*F*(1, 109) = 6.942, *p* =.01
CTQ Physical Neglect	12.03 (4.38)	13.85(5.78)	10.69 (2.2)	*F*(1, 109) = 16.086, *p* <.001
CTQ Sexual Abuse	7.21 (4.62)	8.11(5.46)	6.55 (3.8)	*F*(1, 109) = 3.145, *p* =.079
PCL-5	34.75 (18.31)	44.66(14.39)	27.47 (17.52)	*F*(1, 109) = 30.236, *p* <.001
R-Span Total Correct	59.48 (18.1)	48.47(19.52)	67.56 (11.7)	*F*(1, 109) = 41.197, *p* <.001
BDI-II		25.45(10.21)		
GAD-7			9.08 (5.32)	
QIDS			11.27 (4.5)	

*ELS* = Early life stress, *CTQ* = Childhood Trauma Questionnaire, *PCL-5* = PTSD Checklist for DSM-5, *R-Span *= Reading Span, *BDI-II* = Beck Depression Inventory Version 2, *GAD-7* = Generalized Anxiety Disorder 7, *QIDS* = Quick Inventory of Depressive Symptomatology. In dataset 1, participants identified one race and were administered the BDI-II but not the GAD-7 or QIDS. In dataset 2, participants had the option identify as more than one race and completed the QIDS and GAD-7, but not the BDI-II. The R-Span Total Correct indicates the maximum R-Span items correctly remembered; participants may score between 0 and 84

All participants completed two appointments. First, all participants provided written informed consent and completed clinical interviews and self-report measures. Then, participants completed a WM task while undergoing functional magnetic resonance imaging (fMRI) to evaluate neural activity.

### Self-report measures

All participants completed self-report measures of childhood maltreatment (The Childhood Trauma Questionnaire (CTQ) short form (D. P. Bernstein et al., 2003) and PTSD symptom severity (The PTSD Checklist for DSM-5 (PCL-5) (Spinhoven et al., 2014). Depressive symptom severity was evaluated using differing questionnaires across datasets; participants in dataset 1 completed the Beck Depression Inventory (BDI-II) (Beck et al., 1996) while those in dataset 2 completed The Quick Inventory of Depressive Symptomology (QIDS) (Rush et al., 2003). Additionally self-reported anxiety symptom severity was only evaluated in individuals in dataset 2, using The Generalized Anxiety Disorder (GAD-7) (Lowe et al., 2008) questionnaire.

### fMRI working memory task: reading span task

All participants completed an MRI-compatible R-Span (Bomyea et al., 2018), using a joystick-operated mouse cursor. They were instructed to memorize items while simultaneously solving a secondary processing task in which they decided if a sentence was logically correct as quickly and accurately as possible. The behavioral outcome was total items correctly recalled, with a maximum score of 84 correctly recalled items. See the Supplement (*Reading Span Task Description*) and (Bomyea et al., 2018) for further description and graphical depiction of the task.

### fMRI data acquisition

All participants were scanned in a 3-Tesla scanner using a 32 - channel head array coil. Both datasets were collected at the University of California San Diego Center for Functional Magnetic Resonance Imaging using the same R-Span task. Dataset 1 was collected on a GE Medical systems scanner and Dataset 2 was collected on a Siemens Prisma scanner. Each scanning session included a three-plane scout scan, a sagittally acquired sequence for acquiring T1-weighted images, and one T2*-weighted axially acquired multi-band echo-planar imaging (EPI) scan to measure blood oxygen level dependent (BOLD) signals during the task (see Supplementary Table 1 for scan parameters for each dataset).

### fMRI preprocessing

The imaging data were preprocessed and normalized to Montreal Neurological Institute (MNI) coordinates using tools available in Advanced Normalization Tools Software (ANTsR), (https://github.com/ANTsX/ANTsR), a statistical interface between Analysis of Functional NeuroImages (AFNI) and R software. Correction for susceptibility-induced distortions was done using FSL topup (see (Andersson et al., 2003). Each subject’s T1 images were inhomogeneity-corrected (ANTsR: abpN4) and registered to the *1 mm mni_icbm152_nlin* template (Avants et al., 2011; Manera et al., 2020). EPI data were despiked (AFNI:3dDespike), field bias corrected (ANTS: timeseriesN3), and slice time corrected (AFNI:3dTshift). Motion correction and CompCor denoising (Behzadi et al., 2007), were conducted using ANTsR: preprocessfmri. Images were spatially smoothed using Perona-Malik anisotropic diffusion (Nam et al., 2011) and corrected EPI images were coregistered to the individual standardized T1 image (SyNBold) with forward and inverse registration transformations concatenated and applied in a single step (antsApplyTransforms). EPI data were resampled to 2.4 mm^3^ and converted to z-scores to be used for analysis. Response regressors were created for each phase of the task based on the unique timing of presentation for each phase for each participant. The phases included sentence reading and verification, stimuli encoding, and stimuli recall (see Supplement: *Reading Span Task Description*). Encoding and recall phases were each weighted by set size to model WM load variance. Encoding and recall phases were also weighted by PI control demand to reflect the cumulative increase in PI with consecutive presentation of similar stimuli.

### fMRI task analysis

Voxel-wise activation data were extracted through an a-priori defined WM mask using neuroanatomical atlases of regions relevant to WM (NeuroSynth v5 “Working Memory” Topic 045). Table 2 presents the center of mass coordinates and size for each of the a-priori defined regions of interest (ROIs) in this mask and Figure S1 in the supplement displays the ROI map.

**Table 2:** Center of mass coordinates for regions of interest (ROI) extracted through the NeuroSynth mask to evaluate activation to the R-Span encoding size R0I. See Supplementary Figure S1 for visual depiction of this mask

L IFG	x	y	z	Cluster Size
	−39.4	12.3	36.5	1505
L IPL	−31.5	−56.9	45	1188
R MFG	16.9	10.1	51	1177
R IPL	33.3	−54	44.9	1039
R dlPFC	39.9	35	21.3	958
L dlPFC	−36.3	51.8	10.4	166
L Amygdala	−21.6	−1.7	−17.7	122
R IFG	48.8	8.5	24.6	107
L Insula Lobe	−29.9	22.7	0.2	88

Note. IFG = inferior frontal gyrus; IPL = inferior parietal lobule; MFG = middle frontal gyrus, dlPFC = dorsolateral prefrontal cortex

## Statistical analyses

Behavioral data were analyzed using SPSS (Version 29.0.1.1). Spearman bivariate correlations were used to investigate if CTQ and clinical symptom severity were associated with R-Span Performance. Spearman bivariate correlations were used to identify ROIs that may be most influenced by CM across datasets (FDR corrected at *p* <.01 to control for multiple comparisons of 9 ROIs). Significant correlations between CTQ severity and specific ROIs were followed up with linear regression analyses to examine the unique association between CTQ and neural activity, controlling for psychopathology. The follow-up regression analyses were performed in each dataset independently to investigate if significant unique relationships replicate across datasets. Given the differences in sample inclusion criteria (i.e., dataset 1 included Veterans with PTSD while dataset 2 included individuals with internalizing disorders), the samples were compared on clinical features, age, and sex, using univariate analysis of variance (ANOVA) for continuous measures and Chi-square for categorical measures; Additionally, an ANOVA was utilized to investigate if dataset membership moderated the relationship between CTQ and neural activity.

The continuous CTQ total score was used for the present study’s analyses. Though many studies use CTQ as a continuous measure of childhood maltreatment severity, other similar studies investigating childhood maltreatment have dichotomized individuals into those with and without early life stress (ELS) based on the severity of childhood trauma, according to published cut-off scores for each CTQ subscale (see (D. Bernstein, P. & Fink, 1998; Philip et al., 2013). Therefore, to further integrate the present study’s findings into the existing literature, we present additional analyses and results based on this dichotomization of CTQ severity into an ELS positive and ELS negative category. We used ANOVA and Chi-square to examine differences in demographic, clinical, and neural activity between individuals deemed ELS positive or ELS negative (see supplementary materials, Section 2.2 and supplementary Table 2 for a detailed description of ELS dichotomization, analyses, and results).

## Results

### Participants characteristics and behavioral performance

Demographic, clinical, and task performance data for all participants are presented in Table 1. Most participants identified their race as white (70.3%), their biological sex as female (60%), and reported receiving two or more years of college education (57.6%) (Table 1). Significant differences emerged between datasets (see Table 1): Dataset 1 had a higher proportion of males and participants were older, which is consistent with the higher proportion of males and middle-aged adults that seek VA care relative to the general La Jolla community, which dataset 2 represents (Frueh et al., 2007; Lehavot et al., 2018; Tran, 2017). Similarly, dataset 1 had significantly higher CTQ and PCL-5 scores which is consistent with research suggesting that compared to civilians, Veterans are more likely to have experienced CM (Blosnich et al., 2014; Evans et al., 2018; Schultz et al., 2012) and be diagnosed with PTSD (Trautmann et al., 2017). Additionally, dataset 1 was exclusively comprised of Veterans with a diagnosis of PTSD while dataset 2 included individuals with internalizing disorders broadly. Finally, dataset 2 exhibited better performance on the R-Span. The distribution of performance on the R-Span task across both datasets had slight skewness (= −1.013), normal kurtosis (= 0.936) (Mishra et al., 2019), and no evidence of a ceiling effect (i.e., only 2 participants obtained the maximum score of 84). Associations between behavioral performance on the R-SPAN and CTQ (*r* = −.163, *p* =.046) and PCL (*r* = −.195, *p* =.017) (uncorrected) did not reach statistical significance after controlling for multiple comparisons (FDR corrected at *p* <.01 to control for multiple comparisons). Similarly, R-Span performance was not significantly associated with symptoms of depression or anxiety *p* >.1.

### CM and neural activity

**Fig. 1:**
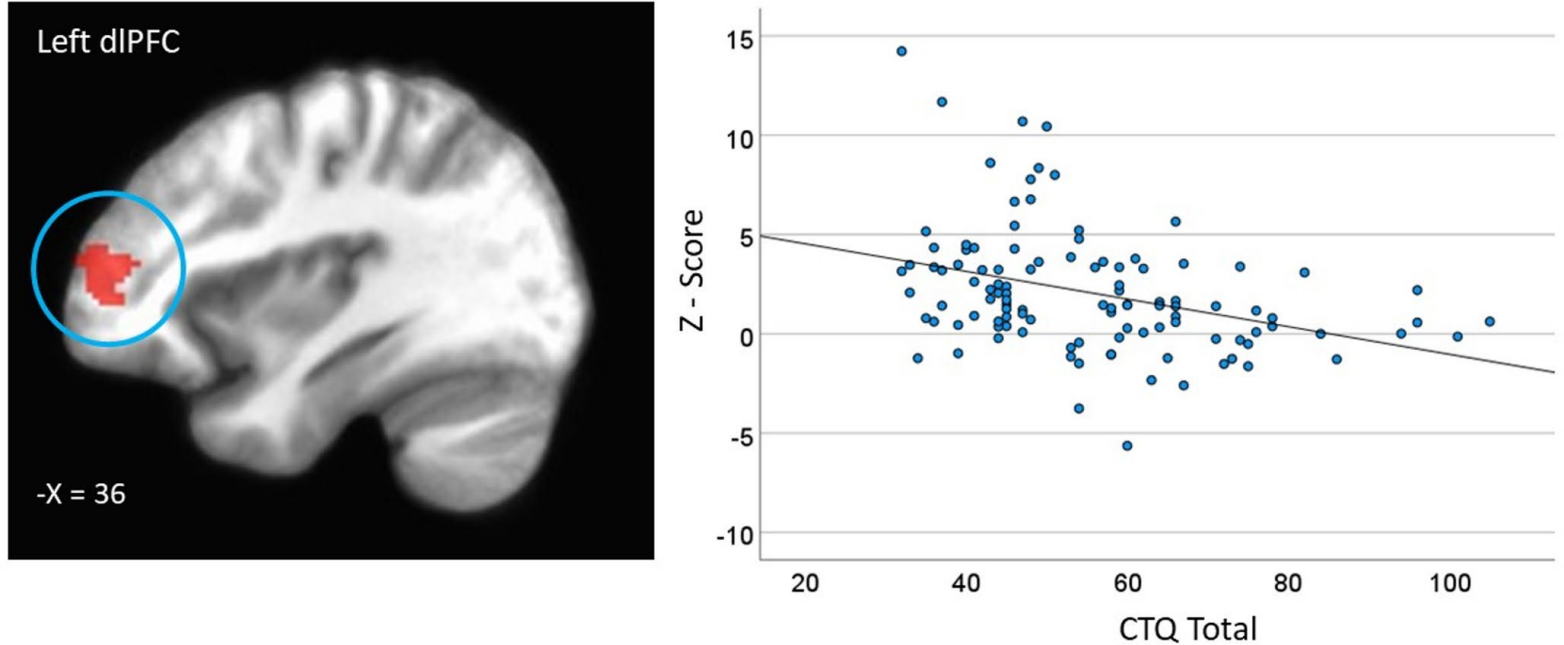
Greater severity of childhood maltreatment (CTQ) is associated with decreased standardized estimates of activation (z-score) in the left dorsolateral prefrontal cortex during the reading span task encoding phase (weighted by set size)

Across both datasets, CM severity (CTQ total) was negatively correlated with activation in the left dorsolateral prefrontal cortex (dlPFC) (*p* <.001) (FDR corrected at *p* <.01 to control for multiple comparisons) (Fig. 1; Supplementary Table 3) during WM encoding (R-Span task) (see Supplementary Table 4 for follow-up linear regressions controlling for dataset, age and sex).

Then a univariate analysis of variance revealed that dataset 1 had significantly more severe CM than dataset 2 (*p* =.002; dataset 1 (*M (SD) =* 61.47 (18.4); dataset 2 (*M (SD) =* 50.16 (13.1)), poorer R-Span performance (*p* <.001, dataset 1 (*M (SD)* = 59.48 (18.1); dataset 2 (*M (SD)* = 48.47 (19.52); however, despite these differences there was no statistically significant interaction between left dlPFC activity and dataset (*p* =.085). This suggests the relationship between left dlPFC activity and CTQ total was not moderated by dataset, thus supporting a generally consistent pattern between CM severity and dlPFC functioning despite the sociodemographic and clinical differences by dataset.

Finally, follow-up linear regression analyses conducted within each dataset, revealed that in dataset 1, CTQ severity was uniquely associated with neural activity in the left dlPFC, controlling for symptoms of depression and PTSD (*ΔR*^*2*^ = 0.11, β = −0.03, *t* = −2.21 *p* =.026) (Table 3); this remained significant when additionally controlling for age and sex (*ΔR*^*2*^ = 0.125, β = −0.024, *t* = −2.025 *p* =.046 (Supplementary Table 5). This finding was then replicated in dataset 2; similarly, CTQ severity was uniquely associated with neural activity in the left dlPFC, controlling for symptoms of depression, and anxiety, (*ΔR*^*2*^= 0.11, β = −0.09, *t* = −2.34, *p* =.023) (Table 4)); this remained significant when additionally controlling for age and sex (*ΔR*^*2*^ = 0.181, β = −0.065, *t* = −172 *p* =.034 (Supplementary Table 6). Both regression analyses revealed that greater CTQ severity was uniquely associated with decreased neural activity in the left dlPFC during the encoding phase of the R-Span task.

**Table 3:** Dataset 1: Childhood trauma severity predicts left middle frontal gyrus activation during the R-Span task, controlling for current symptoms of PTSD and depression

	B	SE	t	*p*	95% CI	
Step 1: *ΔR2* = 0.003; *p* =.94						
BDI-II	−0.010	0.031	−0.321	0.750	−0.072	0.052
PCL-5	0.007	0.022	0.337	0.738	−0.037	0.052
Step 2: *ΔR2* = 0.11; *p* =.026						
CTQ	−0.03	0.013	−2.313	0.026	−0.056	−0.004
BDI-II	−0.004	0.03	−0.131	0.897	−0.064	0.056
PCL-5	0.017	0.021	0.786	0.436	−0.026	0.06

*CTQ* = Childhood Trauma Questionnaire, *PCL-5* = PTSD Checklist for DSM-V, *BDI-II* = Beck Depression Inventory Version 2

**Table 4:** Dataset 2: Childhood trauma severity predicts left middle frontal gyrus activation during the R-Span task, controlling for current symptoms of PTSD, depression, and anxiety

	B	SE	t	*p*	95% CI	
Step 1: *ΔR2* = 0.024; *p* =.475						
QID	0.058	0.131	0.441	0.661	-0.205	0.320
GAD-7	-0.130	0.111	-1.171	0.246	-0.352	0.092
Step 2: *ΔR2 = 0.106; p =.023*						
CTQ	-0.085	0.036	-2.342	0.023	-0.158	-0.012
QID	0.113	0.129	0.874	0.386	-0.145	0.37
GAD-7	-0.098	0.108	-0.902	0.371	-0.314	0.119

*CTQ* = Childhood Trauma Questionnaire, *QIDS* = Quick Inventory of Depressive Symptomatology, *GAD-7* = Generalized Anxiety Disorder 7

## Discussion

The present study examined the extent to which CM was associated with neural activity in frontoparietal circuitry during WM performance within individuals with internalizing disorders. Exposure to more severe CM was associated with decreased neural activity in the left dlPFC across all participants during the R-Span task. Furthermore, this relationship was replicated in both individuals with a diagnosis of PTSD (dataset 1) and individuals with anxiety, mood, and traumatic stress disorders (dataset 2), controlling for current clinical symptoms. CM severity was also associated with poorer WM task performance, however no statistically significant association was observed between left dlPFC activity and task performance. Overall, these findings further understanding of the potential influence of CM exposure on regions vital for WM and suggest that the heterogeneity among individuals with similar diagnoses may be accounted for by CM.

Of the regions examined, only dlPFC activation during WM encoding was associated with CM. The left dlPFC has been implicated in goal-directed attention, reorienting attention, visuospatial processing, and suppressing goal-irrelevant information, thus thought to influence WM capacity and function (Friedman & Robbins, 2022; Japee et al., 2015; Minamoto et al., 2010; Nee et al., 2013; Squire et al., 2013; Xu et al., 2023). Individuals with lower WM capacity have been found to exhibit less dlPFC activation during suppression of task irrelevant stimuli, as well as poorer performance, during a WM task with distractors (Minamoto et al., 2010). Contrary to our expectations, the left dlPFC activation during WM encoding was not significantly associated with WM performance. This lack of association may be due to limited power to detect neural correlates of performance or potentially unaccounted for relationships with neural functioning, such as neural compensation, connectivity, or modulation (Chai, Abd Hamid, & Abdullah, 2018).

The present study suggests that CM exposure severity may be a factor driving variability within clinical populations that is unaccounted for by variability in clinical symptom presentation. There is limited research investigating potential differences in neural functioning within clinical populations with varied CM exposure; the majority of extant research investigating the potential effects of CM on neural functioning during neutral (i.e., without valanced components) WM tasks have primarily included only healthy controls or clinical samples limited by the inclusion of a single diagnosis (e.g., schizophrenia, bipolar disorder (Quide et al., 2017) or MDD (Miller et al., 2015). Our results differ from findings in individuals with MDD, where CM was associated with increased activation in dlPFC (Miller et al., 2015), however, this difference may be partially explained by differences in task design and analysis. However, our findings align with previous studies in healthy controls, suggesting that more severe exposure to CM is associated with decreased frontoparietal activity during WM tasks (Hallowell et al., 2019; Philip et al., 2013, 2016; Raine et al., 2001). The studies in healthy controls tend to use tasks requiring more encoding (i.e., 2-back design) than Miller and colleagues’ (2015) 1-back design, which may account for the similarities to our findings. Overall, we suggest that there may be understudied variability in frontoparietal functioning underlying WM functioning differences within clinical groups.

The present study’s results suggest that CM may increase risk for dysfunction in behavioral and neural indicators of WM ability beyond the effects of psychopathology. Previous research has demonstrated links between psychopathology and poor WM performance (e.g., see (Lukasik et al., 2019; McTeague et al., 2016) as well as neural dysfunction during WM (e.g., PTSD (Aupperle et al., 2012; Moores et al., 2008), anxiety (Balderston et al., 2017; Basten et al., 2011), and MDD (McTeague et al., 2016; Wang et al., 2015)). However, CM is highly prevalent in psychiatric samples and prior work has not sufficiently considered the potential contribution of CM to observed differences between clinical samples and healthy comparators.

CM exposure’s association with altered neural activity in regions implicated in WM may have clinical implications. Executive dysfunction is one factor thought to increase cognitive-based treatment drop-out and decrease treatment response (Crocker et al., 2018; Groves et al., 2018; Moran, 2016). Cognitive-behavioral therapies, considered the gold standard interventions for many psychiatric conditions, utilize cognitively intensive techniques such as completing worksheets to restructure thoughts and feelings. Poor WM may make it difficult for individuals to effectively utilize these techniques and confer benefit from the treatment components (Hronis et al., 2017). Compared to those without CM exposure, individuals with a history of CM have been found to exhibit poorer treatment response to pharmacotherapy, psychotherapy, or combination treatment (Angulo et al., 2024; Lippard & Nemeroff, 2020; Nanni et al., 2012). Variability in treatment response could be related to the potential influence of CM on WM-related processes that are critical to treatment engagement. Further research is necessary to investigate if adjunctive treatments, targeting WM abilities and underlying neural functioning, might improve treatment response to front-line treatments in patients with a history of CM.

Several limitations should be considered while interpreting these findings. Future research with larger sample sizes will be necessary to investigate if this relationship varies across symptom presentations and diagnostic category. Secondly, the present results are replicated across two scanning sites; however, the use of two different MRI scanners may have presented potential confounding effects in the initial selection of ROIs. Given this potentially confounding effect, additional correlations were conducted within each MRI scanner to evaluate consistency of results (see Supplementary Table 7). Third, the present study did not investigate how these relationships varied across sexes. Previous research suggests that sex may play a moderating role on the association between CM and neural functioning during cognitively demanding tasks; however, research is limited and inconsistent regarding if either males or females are neurodevelopmentally more sensitive to CM (White & Kaffman, 2019), with previous studies finding evidence of greater altered neural functioning during cognitive tasks uniquely in males (Crozier et al., 2014) and females (Colich et al., 2017). Research suggests performance on working memory tasks is related to performance on intelligence tests (Verguts & De Boeck, 2002). However, the present study did not include any broader measures of cognitive functioning; thus, future research is necessary to confirm if these observed effects are unique to working memory and remain robust while covarying broader measures of intelligence. Recent research suggests that the relationship between CM and working memory performance differs across ethnic groups (Brown et al., 2022); therefore future research examining these effects neurally is warranted. Additionally, CM was reported retrospectively which may introduce potential biases and we did not assess the age at which CM occurred but this information would provide valuable information about which neural regions were developing at the time of CM exposure. Research suggests that neural regions may be more vulnerable to the effects of stressors during critical periods of development; for example, the PFC’s neurodevelopment may be particularly sensitive to the effects of CM between the ages of 13 and 18 years (Buimer et al., 2022; Cowell et al., 2015; Sheridan et al., 2017; Teicher et al., 2016). The present study examined only the encoding phase of the R-Span task because our prior work indicated that the encoding phase of the R-Span task yields more robust activation as an indicator of working memory interference control than the recall phase (Bomyea et al., 2018) and research suggests that neural activity during the updating phase of working memory differs between individuals with and without childhood maltreatment (Miller et al., 2015). We utilized an a-priori analysis of ROI activity, so the results do not explore CM’s influence on functional connectivity. Research suggests that CM is also associated with widespread changes in functional connectivity, notably increased resting state connectivity between limbic and frontal regions in children and young adults with and without psychopathology (Gerin et al., 2023). Employing analyses of functional connectivity might further understanding of potential widespread changes in neuromodulation, compensatory functioning, or self-referential processing (Schrammen et al., 2022) in the context of psychiatric disorders as well.

## Conclusions

In summary, the present study investigated associations between CM and neural functioning during a WM task, in individuals diagnosed with anxiety, post-traumatic stress, and mood disorders. Greater CM severity was associated with decreased dlPFC neural activity during WM encoding, accounting for current symptoms of psychopathology. The findings further understanding of heterogeneity of neural functioning within clinical populations with and without exposure to CM. Front-line treatments rely on higher-order cognitive skills, such as WM; therefore, individuals with CM might benefit from interventions aiming to enhance WM and prefrontal function.

## Supplementary Information

Below is the link to the electronic supplementary material.

Supplementary Material 1 (DOCX 184 KB)

## Data Availability

The data that support the findings of this study are available on request from the corresponding author. The data are not publicly available due to privacy or ethical restrictions.
