# Supplementary material for: The association between childhood maltreatment and neural functioning during a working memory task in adults with internalizing disorders
Source: Brain Imaging Behav. 2026 Mar 10;20(2):39. doi: 10.1007/s11682-026-01120-2 (PMC12975780; doi:10.1007/s11682-026-01120-2)
Supplement: Supplementary file 1 — Supplementary Material 1 (DOCX 184 KB) [file 11682_2026_1120_MOESM1_ESM.docx]

**The Association between Childhood Maltreatment and Neural Functioning during a Working Memory Task in Adults with Internalizing Disorders**

Morgan M. Caudle, Alan N. Simmons, Reanne C. Moore, Michael L. Thomas, and Jessica Bomyea

**Supplementary Materials**

**1. Participant Inclusion Exclusion Criteria**

The present study utilized data from two datasets. For inclusion in the present analysis, across both datasets, all participants were (1) aged 21 to 55, (2) fluent in English, (3) had outpatient status, if in mental health treatment (4) a current diagnosis of one or more mood, anxiety, or post-traumatic stress disorders, and (5) at least 6-week stability on any prescribed psychotropic medication. Exclusion criteria were (1) past year diagnosis of severe alcohol use or substance disorder, (2) currently receiving evidence-based psychotherapy for mood, anxiety, or traumatic stress disorders, (3) lifetime history of psychotic or bipolar disorder, (4) acute suicidality necessitating immediate clinical intervention, (5) neurodegenerative or neurodevelopmental disorders, (6) history of severe traumatic brain injury (TBI), (7) sensory deficits that would preclude completing tasks, (8) conditions unsafe for completing MRI scanning (e.g., metal in the body; pregnancy) and (9) those with life-threatening or acutely unstable medical conditions. Inclusion criteria were evaluated using the Mini International Neuropsychiatric Interview for DSM-5 (MINI Version 7.0.2) and the PhenX Anxiety Disorders Screener (Composite International Diagnostic Interview Screening Scales (CIDI-SC (Kessler et al., 2013)) or Clinician Administered PTSD Scale for DSM-5 (CAPS-5) interview.

**2. Supplementary Methods and Measures**

***2.1 Reading Span Task Description***

First, during the (1) sentence reading phase: A fixation cross was presented on the screen for 500ms, immediately followed by a sentence (e.g., “Jane walks her car in the park”). Participants were instructed to pull the joystick trigger to indicate that they had finished reading the sentence. After indicating they had read the sentence, a screen with two boxes, one marked “True” and one marked “False” appeared. The participant selected “True” if the sentence was semantically logical or “False” if the sentence was illogical. Then during the (2) encoding phase: The following screen displayed an item (number or letter) for 500ms, which the participant attempted to memorize. Sets of sentences and items continued until the end of the trial. Finally, during the (3) recall phase: The participant was shown a recall screen with twelve items. They were instructed to select the items in the order presented. When the recognition test was complete, the subsequent trial began. Participants received feedback about their sentence accuracy at the end of each trial via a percentage accuracy score. During the instructions, participants were told to aim to keep their accuracy at 85% or above. Spans of 2, 4, and 6 were tested for 7 repetitions each. WM load is thought to increase as to-be-remembered items set sizes increase. The number of to-be-remembered items was presented in a different random order for each set, and sets were presented in a consistent order to participants. Performance on the R-Span relies on the participant’s ability to learn new information during each trial while controlling proactive interference (PI), interference caused by learning from previous trials and sets which contributes to forgetting, and control interference caused by the processing portion of the task (Bunting, 2006; Lustig, May, & Hasher, 2001). Across trials, PI control demands were varied by adjusting the number of consecutive similar to-be-remembered items within and between trials. Repeated presentation of the same category of items has been shown to increase required PI control demands (Bunting, 2006).

***2.2 Early Life Stress and Neural Activity Analysis***

Some similar studies investigating childhood maltreatment dichotomize individuals into those with and without early life stress (ELS) based on the severity of childhood trauma according to published cut-off scores used to determine the severity of each of the five CTQ subscale (i.e., none to minimal, low to moderate, moderate to severe, and severe to extreme (see (Bernstein & Fink, 1998; Philip et al., 2013)). Therefore, individuals scoring either “none to minimal” or “low to moderate” severity across all CTQ subscales were considered ELS negative; Whereas individuals scoring “moderate to severe” or ”severe to extreme” severity on any individual subscale are deemed ELS positive(Philip et al., 2013). These ratings translate numerically, such that, individuals deemed ELS positive are individuals scoring greater than or equal to the published cut-offs scores on any individual subscale (i.e., The cut-off scores for each subscale are: 13 on emotional abuse, 10 on physical abuse, 8 on sexual abuse, 15 on emotional neglect, or 10 on physical neglect)(Bernstein & Fink, 1998). Therefore, to integrate findings with existing literature, we first dichotomized each individual as either ELS negative or ELS positive. Then we explored demographic, clinical, and neural differences between the ELS negative and ELS positive groups using ANOVA. Differences in demographic and clinical measures between those with (n = 65) and without (n = 46) ELS are presented in Supplementary Table 2. Biological sex, age, and R-Span performance did not significantly differ between ELS groups, *p* > .4. Those with a history of ELS exhibited a trend of less activation in the left dlPFC during the R-Span, F(1, 109) = 5.182, p = 0.025, however this difference did not survive correction for multiple comparisons. No other neural differences between groups were observed, p > .05.

***Figure S1.***

*Map of a-priori defined working memory mask.*


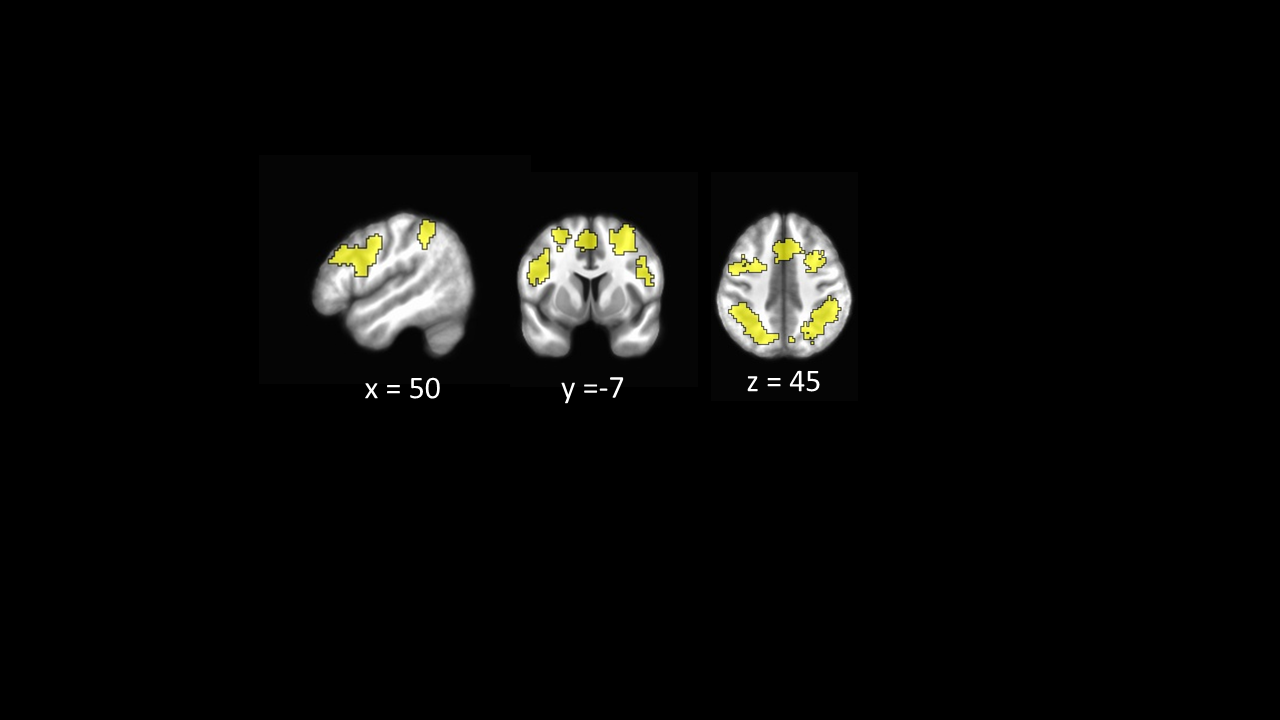


*Note*. Mask defined using neuroanatomical atlases of regions relevant to working memory (NeuroSynth v5 “working memory” Topic 045).

| **Supplementary Table 1** |  |  |
| --- | --- | --- |
| *Scan parameters of both datasets* | |  |
|  | Dataset 1 | Dataset 2 |
| MRI System | GE Medical Systems | Siemens Prisma |
| T1 (MPRAGE) sagittally acquired | |  |
| FOV (cm) | 256x256 | 240x256 |
| Matrix | 256x256 | 300x320 |
| Slices | 208 | 208 |
| Slice thickness (mm) | 1 | 0.8 |
| TR (ms) | 6900 | 2400 |
| TE (ms) | 2.9 | 2.22 |
| Gap (mm) | 0 | 0 |
| Flip angle (degrees) | 8 | 8 |
| T2* |  |  |
| FOV (cm) | 216x216 | 208x208 |
| Acquisition matrix | 90x90 | 104 x 104 |
| TR (ms) | 800 | 800 |
| TE (ms) | 30 | 37 |
| Gap (mm) | 0 | 0 |
| Flip angle (degrees) | 52 | 52 |
| *Note.* T2*-weighted axially acquired multi-band echo-planar imaging (EPI) scan to measure blood oxygen level dependent (BOLD) signals during the task. | | |

**Supplementary Table 2**

| *Demographics and clinical characteristics of ELS positive and ELS negative participants.* | | | | |
| --- | --- | --- | --- | --- |
| Characteristic  Mean (SD) | All Participants  Mean (SD) | ELS Negative  Mean (SD) | ELS Positive  Mean (SD) | Statistic |
| N | 111 | 46 | 65 |  |
| Sex n(% Female) | 65 (59%) | 26 (57%) | 41 (63%) | *χ*2(1) = 0.484, *p* = 0.487 |
| Age | 34.53 (9) | 34.99 (9.39) | 34.21 (8.77) | *F*(1, 109) = 0.201, *p* = 0.655 |
| CTQ | 55.43 (16.02) | 41.85 (5.44) | 65.05 (13.91) | *F*(1, 109) = 115.145, *p* <.001 |
| QIDS | 11.27(4.5) | 12.03 (4.86) | 11.27(4.5) | *F*(1,62) = 1.994, *p* = .163 |
| GAD-7 | 9.08(5.32) | 9.58 (5.93) | 9.08(5.32) | *F*(1, 62) = .592, *p* = .445 |
| BDI-II | 25.45(10.21) | 27.88 (10.6) | 25.45(10.21) | *F*(1, 45) = 6.324, *p* = .016 |
| PCL-5 | 34.75 (18.31) | 30.63 (18.14) | 37.66 (18) | *F*(1, 109) = 4.083, *p* = 0.046 |
| R-Span Total | 59.48 (18.1) | 59.3 (20.81) | 59.6 (16.07) | *F*(1, 109) = 0.007, *p* = 0.933 |
| Note. CTQ = Childhood Trauma Questionnaire; sex = biological sex, PCL-5 = PTSD Checklist for DSM-5, R-Span = Reading Span; BDI-II = Beck Depression Inventory Version 2; GAD-7 = Generalized Anxiety Disorder 7; QIDS = Quick Inventory of Depressive Symptomatology. All continuous measures were analyzed using ANOVA. Sex was analyzed using Chi-square. | | | | |

**Supplementary Table 3**

*Association between Childhood Trauma Severity and Neural Activity across Datasets*

|  | CTQ | Uncorrected p-value | Adjusted FDR p-value | |
| --- | --- | --- | --- | --- |
| L IFG | -.207 | 0.029 | 0.06525 |  |
| L IPL | -0.144 | 0.133 | 0.171 |  |
| R MFG | -.192 | 0.044 | 0.0792 |  |
| R IPL | -0.162 | 0.089 | 0.1335 |  |
| R dlPFC | -.269 | 0.004 | 0.018 |  |
| L dlPFC | -.393 | 0.00001 | 0.0009 |  |
| L Amygdala | 0.003 | 0.975 | 0.975 |  |
| R IFG | -0.128 | 0.179 | 0.201375 |  |
| L Insula | -.227 | 0.016 | 0.048 | |

*Note*. A bivariate Spearman Correlation Analysis was performed and results were FDR corrected at p < .01. The Benjamini – Hochberg procedure was used to derive the adjusted FDR p-values. CTQ = Childhood Trauma Questionnaire; IFG = inferior frontal gyrus; IPL = inferior parietal lobule; MFG = middle frontal gyrus; dlPFC = dorsolateral prefrontal cortex. Spearman bivariate correlations were used to examine associations.

**Supplementary Table 4**

*Across both datasets, childhood trauma severity predicts left middle frontal gyrus activation during the R-Span task, controlling for current age, biological sex, and dataset (i.e., MRI study site). Adjusted R2 = .167, p < .001.*

|  | *B* | SE | *t* | *p* | 95% CI |  |
| --- | --- | --- | --- | --- | --- | --- |
| CTQ | -0.054 | 0.019 | -2.903 | 0.005 | -0.091 | -0.017 |
| Age | 0.050 | 0.031 | 1.587 | 0.115 | -0.012 | 0.111 |
| Sex | -0.131 | 0.629 | -0.208 | 0.836 | -1.377 | 1.116 |
| Dataset | 1.735 | 0.675 | 2.571 | 0.012 | 0.397 | 3.072 |

*Note*. CTQ = Childhood Trauma Questionnaire

**Supplementary Table 5**

*Dataset 1: Childhood trauma severity predicts left middle frontal gyrus activation during the R-Span task, controlling for current symptoms of PTSD and depression, biological sex, and age.*

|  | *B* | SE | *t* | *p* | 95% CI |  |
| --- | --- | --- | --- | --- | --- | --- |
| Step 1: *ΔR2* = 0.034; *p* = 0.826 | | |  |  |  |  |
| PCL-5 | 0.010 | 0.023 | 0.452 | 0.654 | -0.036 | 0.057 |
| BDI-II | -0.018 | 0.033 | -0.553 | 0.583 | -0.085 | 0.049 |
| Sex | 0.197 | 0.480 | 0.409 | 0.684 | -0.773 | 1.166 |
| Age | -0.024 | 0.024 | -1.013 | 0.317 | -0.072 | 0.024 |
| Step 2: *ΔR2* = 0.125; *p* = 0.046 | | |  |  |  |  |
| PCL-5 | 0.018 | 0.022 | 0.789 | 0.435 | -0.028 | 0.063 |
| BDI-II | -0.009 | 0.032 | -0.278 | 0.783 | -0.074 | 0.056 |
| Sex | 0.191 | 0.463 | 0.414 | 0.681 | -0.743 | 1.126 |
| Age | -0.019 | 0.023 | -0.836 | 0.408 | -0.065 | 0.027 |
| CTQ | -0.024 | 0.012 | -2.058 | 0.046 | -0.048 | 0 |

*Note. CTQ = Childhood Trauma Questionnaire; PCL-5 = PTSD Checklist for DSM-V, BDI-II = Beck Depression Inventory Version 2*

**Supplementary Table 6**

*Dataset 2: Childhood trauma severity predicts left middle frontal gyrus activation during the R-Span task, controlling for current symptoms of PTSD, depression, anxiety, age, and biological sex.*

|  | *B* | SE | *t* | *p* | 95% CI |  |
| --- | --- | --- | --- | --- | --- | --- |
| Step 1: *ΔR2* = 0.115; *p* = 0.12 | | |  |  |  |  |
| QID | 0.085 | 0.128 | 0.663 | 0.510 | -0.171 | 0.341 |
| GAD-7 | -0.181 | 0.110 | -1.640 | 0.106 | -0.401 | 0.040 |
| Age | 0.122 | 0.055 | 2.232 | 0.029 | 0.013 | 0.231 |
| Sex | -1.157 | 1.106 | -1.046 | 0.300 | -3.371 | 1.056 |
| Step 2: *ΔR2* = 0.181; *p* = 0.034 | | |  |  |  |  |
| QID | 0.135 | 0.126 | 1.071 | 0.288 | -0.117 | 0.388 |
| GAD-7 | -0.16 | 0.107 | -1.49 | 0.142 | -0.375 | 0.055 |
| Age | 0.127 | 0.053 | 2.396 | 0.02 | 0.021 | 0.233 |
| Sex | -0.468 | 1.119 | -0.418 | 0.677 | -2.708 | 1.772 |
| CTQ | -0.065 | 0.03 | -2.172 | 0.034 | -0.125 | -0.005 |

*Note. CTQ = Childhood Trauma Questionnaire; QIDS = Quick Inventory of Depressive Symptomatology; GAD-7 = Generalized Anxiety Disorder 7*

Supplementary Table 7

*Exploratory spearman correlation analysis examining the association between Childhood Trauma Severity and Left Middle Frontal Gyrus Activity within each dataset (i.e., each MRI scanner).*

|  | Dataset 1 CTQ | Dataset 1  *p*-value | Dataset 1 FDR Adjusted *p*-value | Dataset 2 CTQ | Dataset 2  *p*-value | Dataset 2  FDR Adjusted *p*-value |
| --- | --- | --- | --- | --- | --- | --- |
| L IFG | -0.099 | 0.508 | 0.743 | -0.119 | 0.348 | 0.626 |
| L IPL | -0.066 | 0.66 | 0.743 | -0.021 | 0.87 | 0.894 |
| R MFG | -0.277 | 0.06 | 0.27 | 0.088 | 0.488 | 0.732 |
| R IPL | -0.082 | 0.582 | 0.743 | -0.03 | 0.813 | 0.894 |
| R dlPFC | -0.208 | 0.16 | 0.743 | -0.165 | 0.194 | 0.626 |
| **L dlPFC** | **-.347** | **0.017** | **0.153** | **-.295** | **0.018** | **0.162** |
| L Amygdala | -0.013 | 0.93 | 0.153 | 0.136 | 0.283 | 0.626 |
| R IFG | -0.084 | 0.577 | 0.743 | -0.017 | 0.894 | 0.894 |
| L Insula | 0.086 | 0.563 | 0.743 | -0.14 | 0.272 | 0.626 |
| Note. Bivariate Spearman Correlation Analysis; FDR correction applied using Benjamini-Hochberg Adjusted P-value (p <.05); CTQ = Childhood Trauma Questionnaire IFG = inferior frontal gyrus; IPL = inferior parietal lobule; MFG = middle frontal gyrus; dlPFC = dorsolateral prefrontal cortex. Spearman bivariate correlations were used to examine associations. | | | | | | |

**References**

Bernstein, D., P., & Fink, L. (1998). *Childhood trauma questionnaire : a retrospective self-report : manual*. Orlando: Psychological Corporation.

Bunting, M. (2006). Proactive interference and item similarity in working memory. *J Exp Psychol Learn Mem Cogn, 32*(2), 183-196. doi:10.1037/0278-7393.32.2.183

Kessler, R. C., Calabrese, J. R., Farley, P. A., Gruber, M. J., Jewell, M. A., Katon, W., . . . Wittchen, H. U. (2013). Composite International Diagnostic Interview screening scales for DSM-IV anxiety and mood disorders. *Psychol Med, 43*(8), 1625-1637. doi:10.1017/S0033291712002334

Lustig, C., May, C. P., & Hasher, L. (2001). Working memory span and the role of proactive interference. *J Exp Psychol Gen, 130*(2), 199-207. doi:10.1037//0096-3445.130.2.199

Philip, N. S., Sweet, L. H., Tyrka, A. R., Price, L. H., Carpenter, L. L., Kuras, Y. I., . . . Niaura, R. S. (2013). Early life stress is associated with greater default network deactivation during working memory in healthy controls: a preliminary report. *Brain Imaging Behav, 7*(2), 204-212. doi:10.1007/s11682-012-9216-x
